# Decoding hind limb kinematics from neuronal activity of the dorsal horn neurons using multiple level learning algorithm

**DOI:** 10.1038/s41598-017-18971-x

**Published:** 2018-01-12

**Authors:** Hamed Yeganegi, Yaser Fathi, Abbas Erfanian

**Affiliations:** Department of Biomedical Engineering, School of electrical engineering, Iran Neural Technology Research Center, Iran University of Science and Technology (IUST), Tehran, Iran

## Abstract

Decoding continuous hind limb joint angles from sensory recordings of neural system provides a feedback for closed-loop control of hind limb movement using functional electrical stimulation. So far, many attempts have been done to extract sensory information from dorsal root ganglia and sensory nerves. In this work, we examine decoding joint angles trajectories from the single-electrode extracellular recording of dorsal horn gray matter of the spinal cord during passive limb movement in anesthetized cats. In this study, a processing framework based on ensemble learning approach is propose to combine firing rate (FR) and interspike interval (ISI) information of the neuronal activity. For this purpose, a stacked generalization approach based on recurrent neural network is proposed to enhance decoding accuracy of the movement kinematics. The results show that the high precision neural decoding of limb movement can be achieved even with a single electrode implanted in the spinal cord gray matter.

## Introduction

Restoration of paralyzed extremities through functional electrical stimulation (FES) is a presented paradigm for individuals with neuromuscular disorders and spinal cord lesions. In FES methodology, by applying electrical stimulations to cause contractions in the paralyzed muscles and restoring some mobility, not only an improvement in the independence of disabled people is attained but also their general health conditions are ameliorated^1^. In order to take advantage of the benefits of a closed loop scheme in controlling complex limb movements, a continuous feedback of limb states is needed. Cutaneous and proprioceptive afferents have been proposed as a natural source of sensory feedback for FES systems^2^.

Stein *et al*. investigated the possibility of extracting the position of the foot in space (positions and velocities in Cartesian (*x*, *y*) and polar coordinates) from populations of neurons in the dorsal root ganglion (DRG)^3^. For this purpose, they recorded neural signals from up to 100 discriminable nerve cells in the L6 and L7 DRG of the anesthetized cat and employed a linear filter to decode the end-point of the limb in space from the firing rates (FRs) of the sorted neurons. It was reported that predictions using only the one neuron, whose firing was best correlated to the kinematic variable, accounted for about 70% of the variance and it was demonstrated that as more neurons were added, the performance increased and reached a plateau. To find the most informative neurons from a large population of identified neurons, a heavy offline processing is required. This makes the approach difficult for real-time control applications. Moreover, the decoding is based on a linear model. However, the firing rates of individual neurons do not necessarily need to be linearly related to the kinematics^4,5^.

Decoding the hind limb kinematics (i.e., ankle, knee, and hip joint angles) from the neural activity of a few neurons in the L7 dorsal root ganglia of three cats has been investigated during walking using a linear filter^2,6^. To improve the decoding performance, a nonlinear state space model was also employed for decoding the FRs in an ensemble of populations of primary afferent neurons during passive movements^4^. In^5^ a neuro-fuzzy neural network (FNN) was applied for decoding DRG recordings to estimate limb kinematics during passive as well as voluntary limb movements in cats. It was demonstrated that FNN model provided more accurate estimates of limb state and generalized better than multiple linear regression methods. Reconstruction of forelimb kinematic variables from neural activity of DRG neurons has been also studied in monkeys during voluntary reach-to-grasp movements using sparse linear regression analysis^7^. The sensory information including distance and tilt of the vector between hip and limb endpoint, extracted from DRG neurons, has been utilized in the closed-loop control of hind limb movements using functional electrical stimulations^8,9^. Recently, the hind limb states (i.e., knee and ankle angles) were estimated using a dynamic driven recurrent neural network (RNN) from neural activity recorded by a 16-channel single-shank electrode array implanted in L7 DRG. The results show the superiority of a dynamic driven recurrent neural network (RNN) over linear dynamic models^10^. The tactile afferent signals recorded from DRG has been also decoded as different sensory events which are generated by mechanical stimulation of three different areas of the left hind paw, using multilayer perceptron classifier^11^.

All of the aforementioned works investigated extracting kinematic information from DRG neurons. However, it was reported that long-term chronic recordings from DRGs do not last for more than three weeks^2^. This is mostly because of the suspended structure of dorsal root ganglia, and the fact that attaching an array to it would increase the risk of tearing the roots. On the other side, dorsal horn is a compact bulky tissue suggesting a better choice for chronic implantations.

In addition to DRG, the modulation of dorsal spinocerebellar responses to the limb movement has been investigated in^12–15^. It was demonstrated that the activity of dorsal spinocerebellar neurons relates to global parameters of limb movement and posture rather than to specific muscle or joint parameters, specifically to a kinematic representation of the limb endpoint.

The possibility of decoding motor commands from peripheral nerve signals was also investigated^16^. For this purpose, the intra-fascicular electrodes were implanted in the median and ulnar nerves of an amputee’s stump and different hand movements including palmar grasp, pinch grasp, and flexion of the little finger were tried to identify using peripheral nerve signals. Recently, the peripheral nervous system responses to mechanical stimulation of the limb were also investigated^17^. Three types of mechanical stimulations, namely, proprioception, touch and nociception were delivered to the limb and the electroneurogram signals were recorded simultaneously from the sciatic nerve with a 16-contact cuff electrode. The results show that neural responses can be separated according to stimulus type as well as intensity.

A question that arises is whether the kinematic information of hind limb can be extracted from the dorsal horn of spinal gray matter neurons. This is the principle issue to be investigated in this paper. The sensory signals recorded from dorsal horn of spinal cord have been used previously for detecting the sensory events generated by electrical stimulation^18^ and decoding intravesical pressure in rat^19^.

The major focus of the current study is the decoding the hind limb kinematics from extracellular neural activity recorded directly from spinal cord gray matter neurons in the anesthetized cats. Both firing rate (FR) and interspike interval (ISI) were used to estimate the kinematic information. A stacked modular neural network based on the recurrent neural network is proposed as a combining tool for utilizing both FR and ISI information to decode the neural activity and the results are compared with that obtained using the conventional recurrent neural network. Moreover, the effects of multiunit activities (MUAs) and single unit activities (SUAs) on decoding performance are evaluated.

## Methods

### Animal preparation

**Figure 1:**
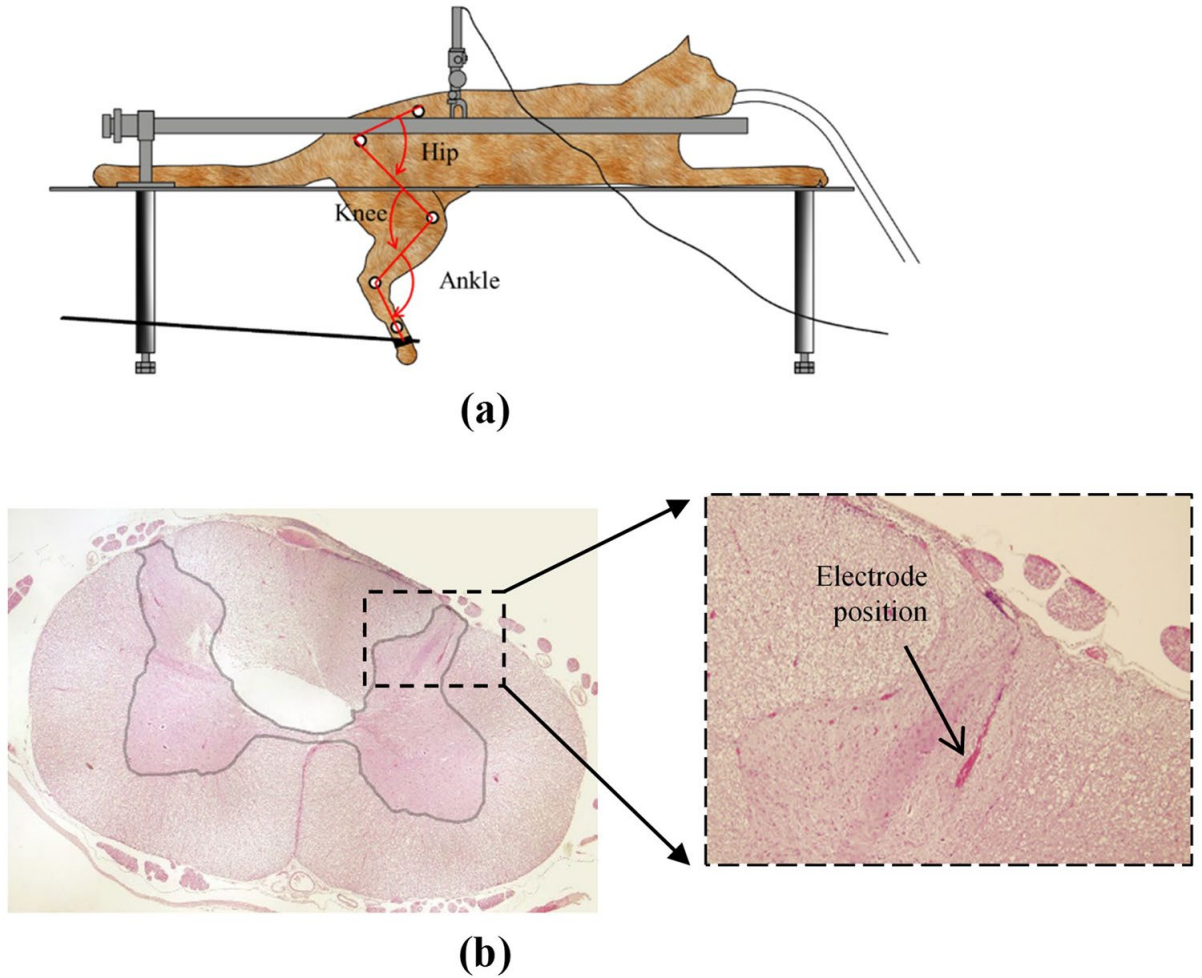
Experimental setup and recording site. (**a**) Hip, knee, and ankle joint angles were calculated having the positions of five markers placed over iliac crest, hip, knee, ankle, and metatarsophalangeal (MTP) joints. A rod was stuck to MTP joint to move the hind limb passively. (**b**) Histology: L6 spinal cord section of cat 5 stained with Hematoxylin and Eosin (H&E) staining.

Five adult cats were used in the present study (3.3 to 3.9 Kg). All surgical procedures and experimental protocols involving animal models described in this paper were approved by the Animal Care and Ethics Committee of Iran Neural Technology Research Centre, Iran University of Science and Technology. The experimental protocol was performed in accordance with the recommendations for the care and use of laboratory animals. The animals were initially anesthetized with ketamine (20 mg/kg) injected intramuscularly into the cranial thigh muscle. The animals were then intubated and maintained at a surgical level of anesthesia with isoflurane (1.0%–3.0% in O2). Blood oxygen saturation level (SpO2) and heart rate signals were monitored continuously throughout the surgical process and experimental tests. A partial dorsal laminectomy was performed to expose L6 up to L3 segments and the dura mater of the dorsal surface of the spinal cord was opened with iridectomy scissors and the spinal cord was covered with saline to prevent its dehydration. The cats were then positioned in a stereotaxic setup (SN-1N, Narishige Group Product) which allows the hind limbs to hang free while the spinal vertebrae (L2 and L7) are clamped rigidly to the frame (Fig. 1(a)).

### Joint angle measurement

The movements were recorded with a motion capture system (Vicon Motion Systems Ltd., UK) with three camera. Reflective markers were attached overlying iliac crest, greater trochanter (hip joint), lateral condyle of the femur (knee joint), lateral malleolus (ankle joint), and the distal end of the fifth metatarsophalangeal (MTP) joint of the cats. The temporal sampling rate was 100 Hz. During each trial of the experiment, an operator moved the foot of the cat in a stepping-like pattern. Having the distance between marker positions, hip, knee, and ankle angles were extracted using the law of cosines. Each recording session consisted of 10 trials of experiment and each recording trial lasted for 5 minutes.

### Neural Data Acquisition and Preprocessing

A single-wire steel electrode with a 75 *μm* shank diameter, Epoxylite insulated with a 10°–5° tapered tip of 120 *μm* exposed length and 300–500 *k*Ω resistance (FHC Inc., Bowdoin, ME USA) was positioned at a location within the right dorsal horn where the correlation of neural activity with passive movement of the limb was visually inspected. The microelectrode was mounted on a micromanipulator (SM-15, NARISHIGE Group Product) that could control the three-dimensional positioning of the electrode with the minimum graduation of 10 µm. The electrode was positioned at the locations within the L6 and L5 dorsal horn, approximately 1–2 mm lateral from the midline between 0.5 and 1.0 mm in depth (Fig. 1(b)). To determine the best electrode position within the dorsal horn, the electrode was vertically advanced through the spinal cord dorsoventrally. Along the electrode track, operator moved the foot of the cat. Then, the electrode was withdrawn and moved 100 μm mediolaterally and/or rostrocaudally to an adjacent location while the correlation of neural activity with the passive movement of the limb was visually inspected on the monitor of the recording system. The positions that produced relatively highest correlation were selected. Neural signals were recorded at 20 kHz sampling rate using a digital data acquisition system (USB-ME64 system, Multichannel Systems Reutlingen, Germany) during the passive movement of the limb.

### Preprocessing and Feature Extraction

The recorded neural signals were band-pass filtered between 300 and 3000 Hz with the low-pass and high-pass elliptic filters of order four. The threshold for spike detection was set to four times the standard deviation of the noise estimated from filtered signal, and spike events were identified as each instance the signal exceeded this threshold. Spikes were sorted using an unsupervised algorithm, Wave_Clus program, that automatically determines the number of classes and assigns each spike to one class based on wavelet coefficients of spike waveforms as the features and superparamagnetic clustering algorithm^20^. The original MATLAB codes are provided by Dr. Quian Quiroga and are publicly available online (http://www2.le.ac.uk/centres/csn/research-2/spike-sorting). The feature set was formed from the continuous firing rate (FR) and the interspike interval variability (ISI).

#### Firing Rate

Continuous FR was computed by taking a window of duration 300 ms and sliding it along the spike train with 250 ms overlap and counting the number of spikes within the window at each time and thereafter low-pass filtering at cutoff frequency of 10 Hz (FIR filter with maximum passband ripple of 1 dB and 60 dB of stopband attenuation). FRs were calculated for either of unsorted and sorted spike trains.

#### Interspike Interval Variability

Consecutive spike peaks constitute an S-S interval time series *i.e*. the ISI signal. S-S interval time series is not sampled at uniform intervals due to differences between the duration of adjacent spikes. Uniform sampling can be performed by using different interpolation methods in order to achieve equally spaced S-S intervals. In this work, the extracted S-S interval time series were interpolated by a cubic spline and then sampled at a rate of 20 Hz.

## Decoding Model

### Recurrent Neural Network

**Figure 2:**
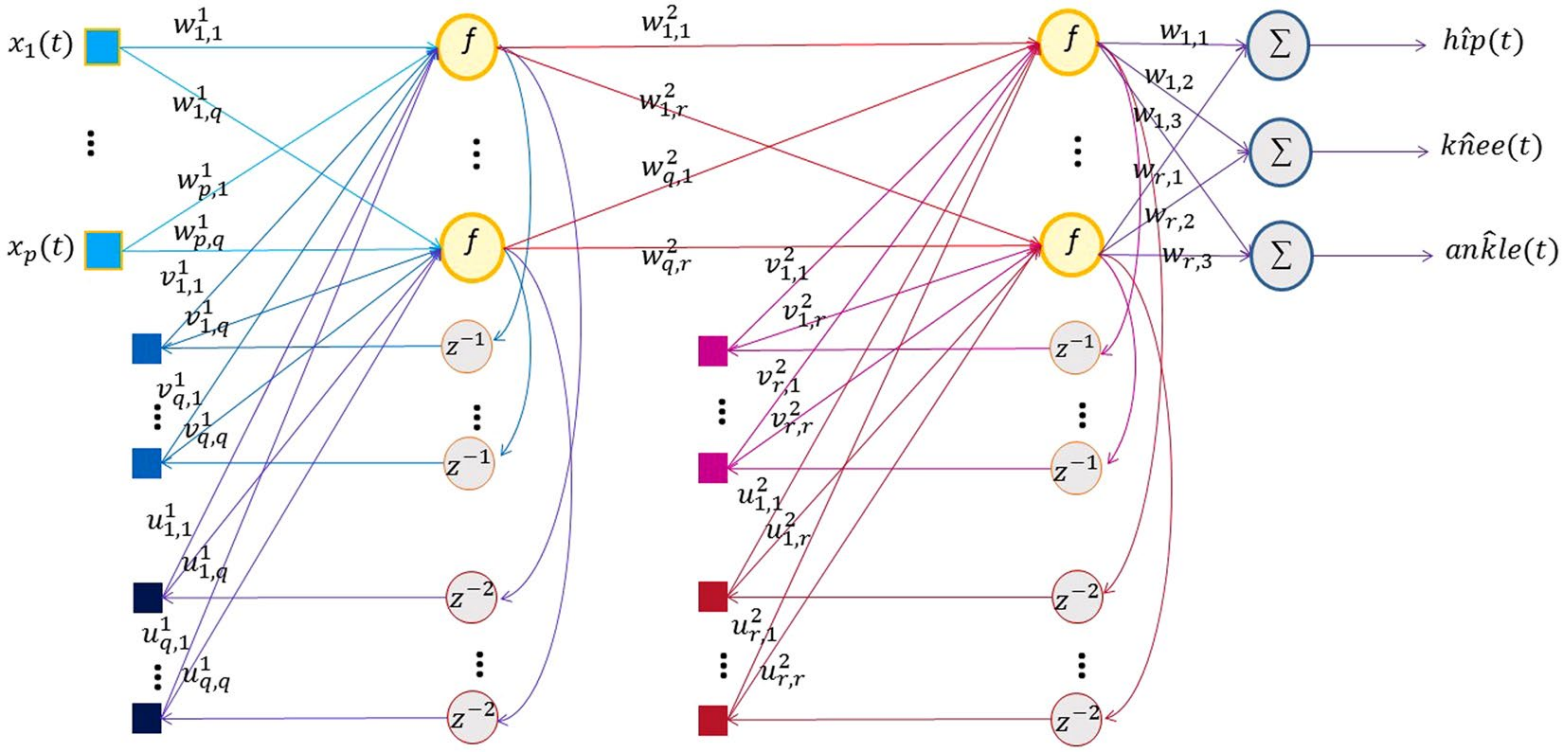
Structure of two hidden-layer recurrent neural network.

The RNN which involves dynamics elements in the form of feedback loop, has a profound impact on the learning capability of the network and on its performance^21^. Moreover, the feedback loops which feedback the lagged outputs of the neurons to the inputs of neurons, enable the network to perform dynamic mapping and learning tasks that extend over the time. The architecture of the RNN takes many different forms^21^. In this work, we use recurrent multilayer perceptron with two hidden layers, as illustrated in Fig. 2(c). The network contains delayed recurrent connections from the output of each hidden layer to its input. We may then describe the dynamic behavior of the network by the following equations:

First hidden layer:

1 $${y}_{i}^{1}(t)=f(\sum _{j=1}^{p}{{w}_{j,i}}^{1}{x}_{j}(t)+\sum _{j=1}^{q}{{v}_{j,i}}^{1}{y}_{j}^{1}(t-1)+\sum _{j=1}^{q}{{u}_{j,i}}^{1}{{y}_{j}}^{1}(t-2))\,(1\le i\le q)$$

Second hidden layer:

2 $${y}_{i}^{2}(t)=f(\sum _{j=1}^{q}{{w}_{j,i}}^{2}{y}_{j}^{1}(t)+\sum _{j=1}^{r}{{v}_{j,i}}^{2}{y}_{j}^{2}(t-1)+\sum _{j=1}^{r}{{u}_{j,i}}^{2}{{y}_{j}}^{2}(t-2))\,(1\le i\le r)$$

Output layer:

3 $$h\hat{i}p(t)=\sum _{j=1}^{r}{c}_{j,1}\,{y}_{j}^{2}(t),k\hat{n}ee(t)=\sum _{j=1}^{r}{c}_{j,2}\,{y}_{j}^{2}(t),an\hat{k}le(t)=\sum _{j=1}^{r}{c}_{j,3}{y}_{j}^{2}(t),$$

where *f*(.,.) is a nonlinear activation function (sigmoid activation function) characterizing the hidden units, ${y}_{i}^{h}(t)$ is the response of the *i*th hidden unit in the hidden layer *h*, ${w}_{i,q}^{l}$ is the connecting weight of unit *i* in layer *l* to the unit *q* in the next layer. The *v* and *u* are the connecting weights of the unit-delay units to the hidden units in the first and second hidden layer, respectively. The hidden layers are nonlinear but the output layer is linear. The RNN is trained using the Levenberg–Marquardt method. The Levenberg–Marquardt method is a compromise between the Gradient descent which has a guaranteed convergence upon a proper choice of the step-size and Newton’s method, which converges speedily near a local or global minimum^22^.

### Stacked Recurrent Neural Network

**Figure 3:**
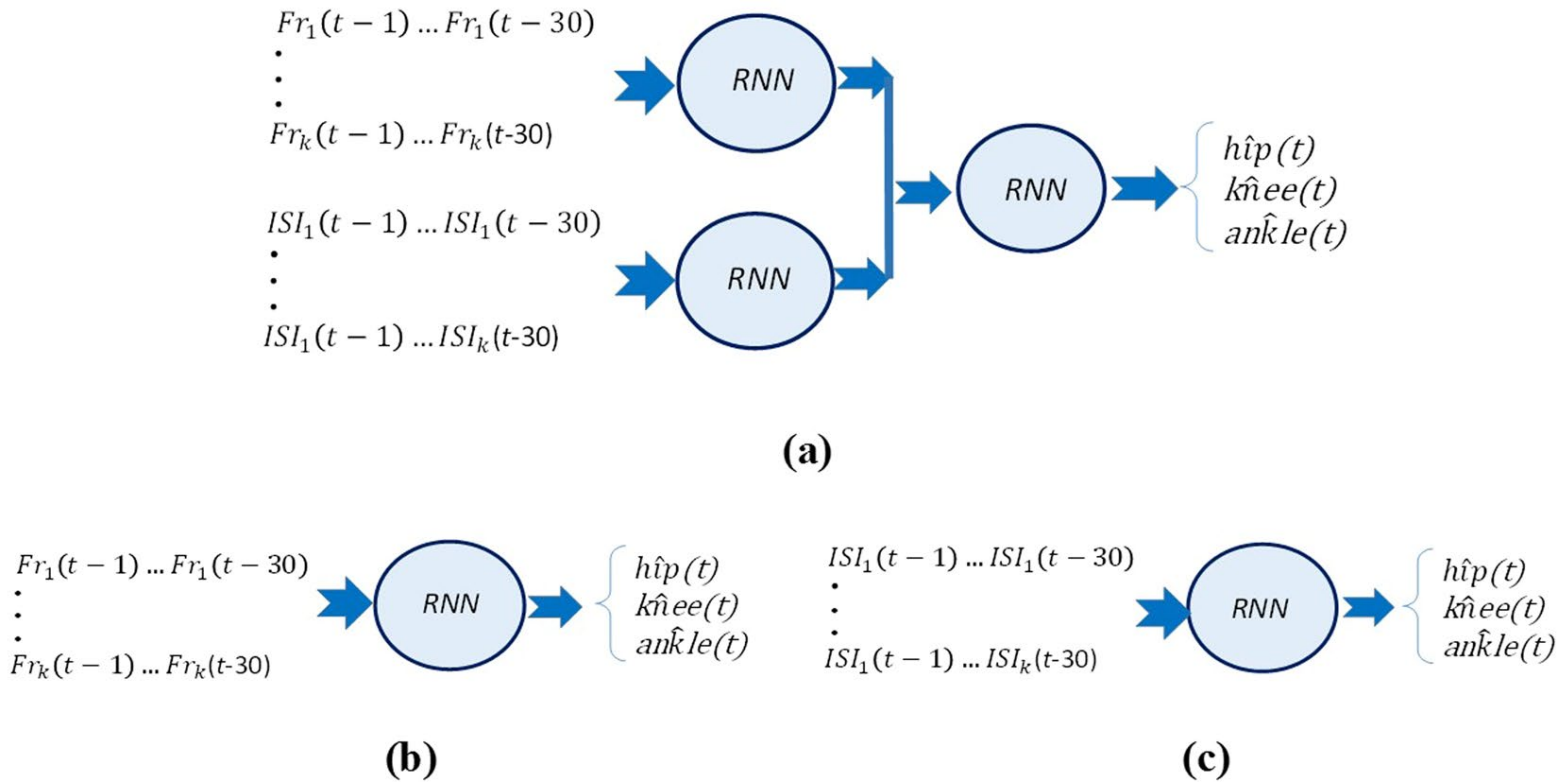
(**a**) Stacked structure. (**b**) The RNN with FRs as the input. (**c**) The RNN with ISIs as the input.

The structure of the proposed stacked RNN is shown in Fig. 3(a). Stacking benefits a two-level learning paradigm. Selected input features are fed into first level models, commonly called level-0 models, and each one produces a prediction value for each output. Then, the outputs of level-0 models are fed into the second stage, or level-1 models, which combines them into the final prediction.

In this work, we take advantage of stacked RNN to fuse continuous ISI and spike related information (i.e., FR). In this way, two RNNs are assigned in the level-0 stage, one for ISI and the other for FR. The past values of the FR and ISI were fed into the RNNs.

All training samples are divided into two parts. The first part is used to train each RNN of the level-0 stage. The second part is used to train the level-1 model using predicted values of level-0 RNNs as the input. Dividing the training samples into two parts and using different training data to train the level-0 and level-1 models may cause an increase of generalization ability of the models^23,24^. Moreover, two different conventional structures were also used to decode joint angles (Fig. 3(b,c)) and the results were compared with stacked structures.

### Data availability

All datasets generated during the current study are available from the corresponding author under request.

## Results

To assess the performance of proposed method in decoding the kinematic information of the limb, the normalized root-mean-square (NRMS) of the estimation error and the coefficient of determination, *R*^2^ value, were used. The NRMS and *R*^2^ were defined as

4 $$NRMS=\frac{1}{{\rm{\max }}(x)-\,{\rm{\min }}(x)}\times \sqrt{\frac{{\sum }_{n=1}^{N}{(x(n)-\hat{x}(n))}^{2}}{N}}\times 100$$

5 $${R}^{2}=(1-\frac{{\sum }_{n=1}^{N}{(x(n)-\hat{x}(n))}^{2}}{{\sum }_{n=1}^{N}{(x(n)-\bar{x})}^{2}})\times 100\,$$

where *x* is the measured joint angle, $\bar{x}$ is the corresponding mean value, $\hat{x}$ is the predicted value, and N is number of data points.

We recorded 10 sets of 5-minute long trials for each animal. The trials where the marker positions had spontaneous jumps were not considered for further analysis. The data set consisted of at least 6 sets of 5-minute-long recording trials. After removing trials with unstable marker positions, from all of the data, two trials were taken apart to be used only for model training, validation and parameter settings (i.e. setting window length of FR, number of neurons in the model, and number of delays).

**Figure 4:**
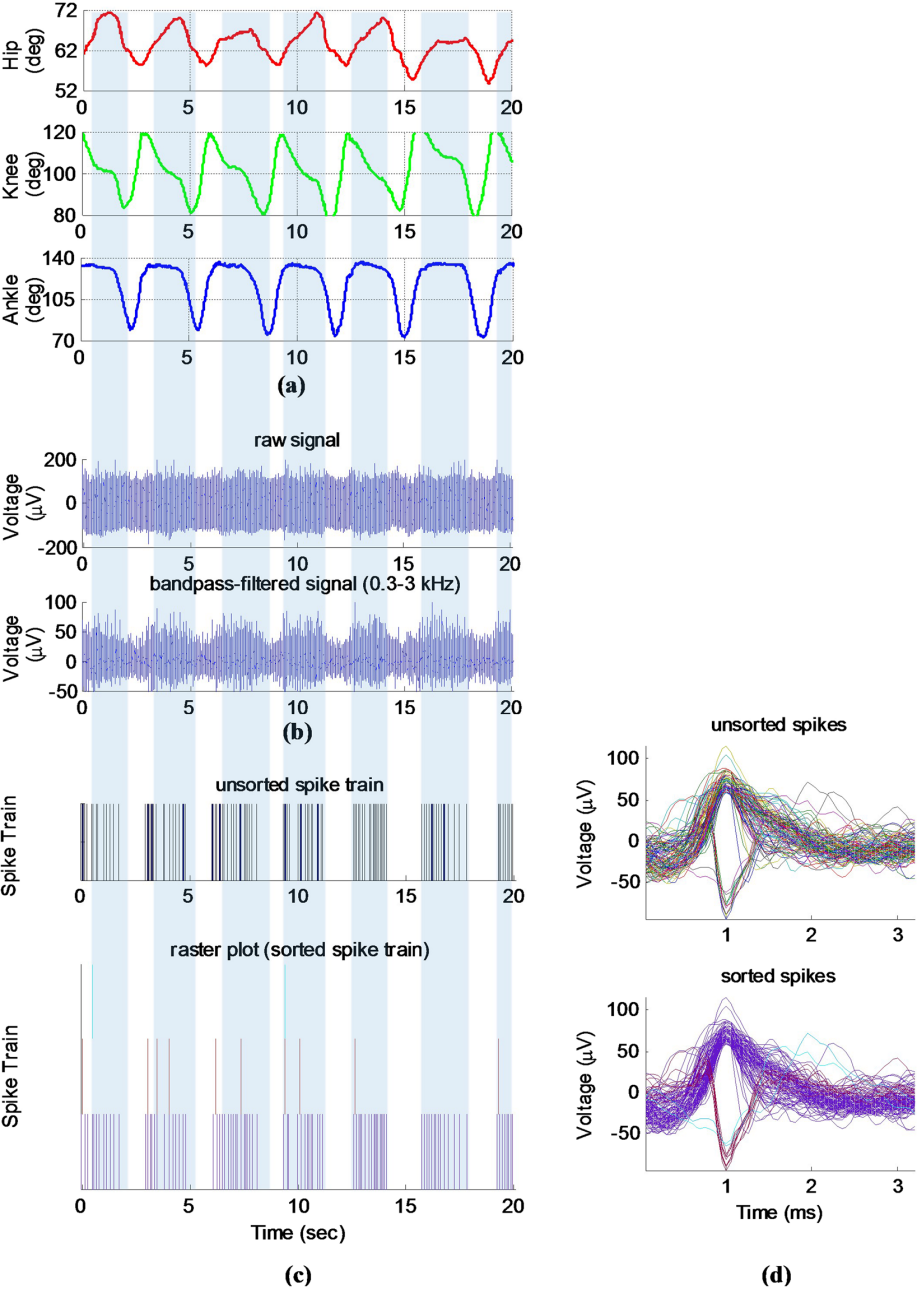
A typical measured joint angles and neural signal recorded from the dorsal horn neurons during passive movement (trial 3, cat 1): (**a**) measured joint angles, (**b**) raw recorded and filtered neural signal, (**c**) unsorted and sorted spike trains, and (**d**) unsorted and sorted spike waveforms.

Figure 4 shows a typical recorded joint angles, raw recorded neural signal, unsorted and sorted spike trains, and the multiunit spike waveforms. It can be seen that the filtered neural signal (as well as the spike trains) is highly correlated with the limb movement. The limb movements as stimuli, give rise to a correlated activity in the spinal cord gray matter neurons. This indicates that reverse regression can be employed for decoding neural responses to estimate limb kinematics.

The results of spike sorting is shown in Fig. 4. A total of 4 units were discriminated from one channel recording on the third trial of experiment on cat 1. One unit has fired as low as one spike per minute and has not fired during 20-s of data shown if Fig. 4(e). This unit is ignored as it may represent spontaneously bursting activity of the neurons in spinal cord. It is observed that among these four units, only the firing of one unit closely correlated with the limb movement.

## ISI and FR

**Figure 5:**
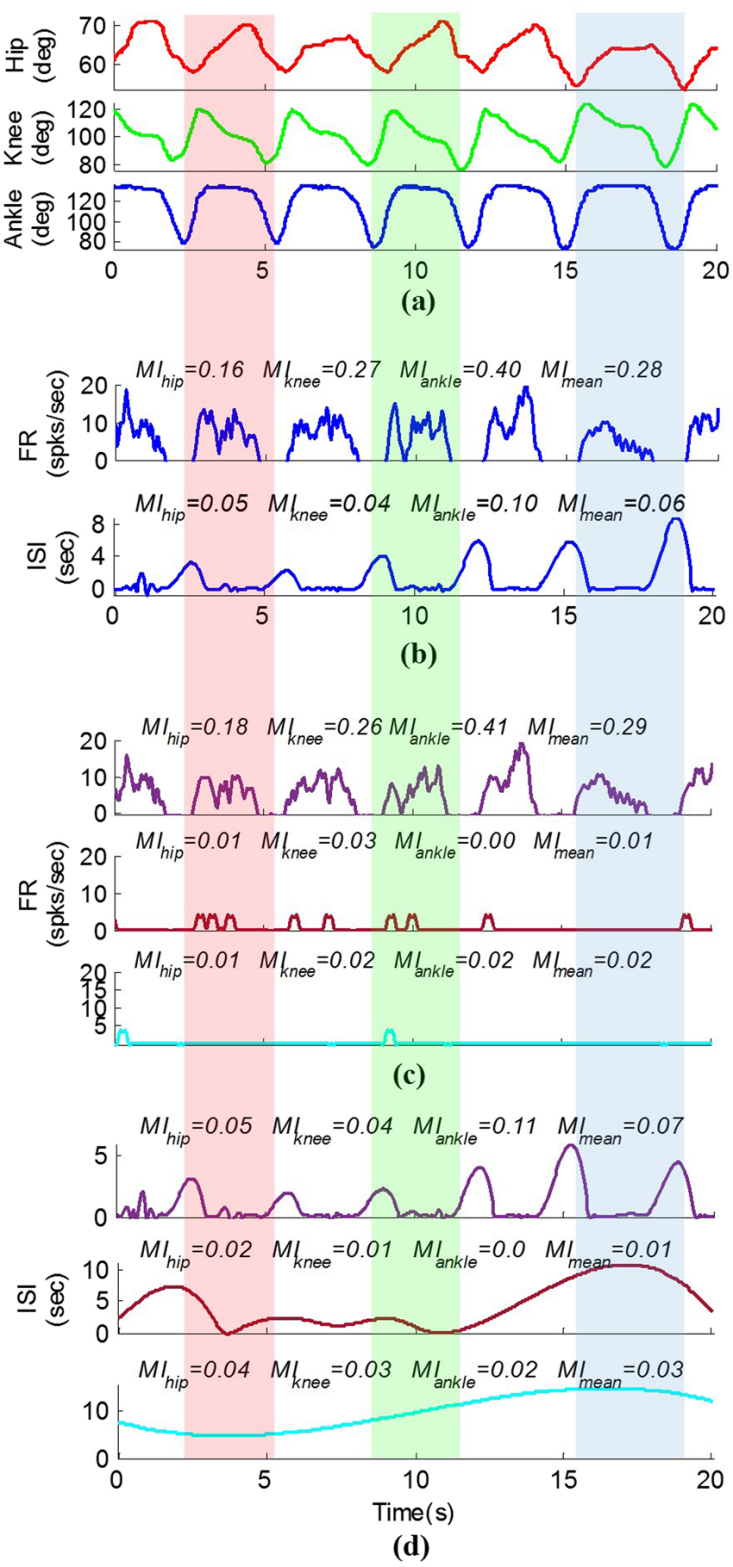
A typical measured joint angles and corresponding continuous firing rate (FR) and interspike interval (ISI) variability during passive movement (trial 3, cat 1): (**a**) measured joint angles, (**b**) multiunit firing rate and interspike interval, (**c**) firing rate of the three identified sorted units, and (**d**) interspike interval of the three identified sorted units.

The results show that the continuous firing rate trajectory closely follows the joint angle variations. Continuous firing rate increases with the extension of the lower limb joints and decreases with the flexion. Figure 5 shows a typical recorded joint angles and corresponding continuous FR and S-S interval time series for unsorted and sorted spike trains. It is observed that increasing the firing rate corresponds to the extension of the hip joint, flexion of the knee joint, and full extension of the ankle. It is observed that the firing activity was diminished during some portions of the movement (i.e., during hip flexion, knee extension, and flexion and extension of the ankle). However, continuous ISI conveys some information about these portions of the movement. The mutual information (MI) between the multiunit FR and the hip, knee, and ankle angles are 0.16, 0.27, and 0.40, respectively and the MI between multiunit ISI and the joint angles are 0.05, 0.04, and 0.10. The results indicate that in addition to FR, the ISI conveys information about the limb movement but not as much as FR.

The results of spike sorting during passive limb movement are shown in Fig. 5(b) and (c). It is observed that only one identified unit could provide information about the limb movement. The MI between the single-unit FR and the hip, knee, and ankle angles are 0.18, 0.26, and 0.41, respectively.

**Figure 6:**
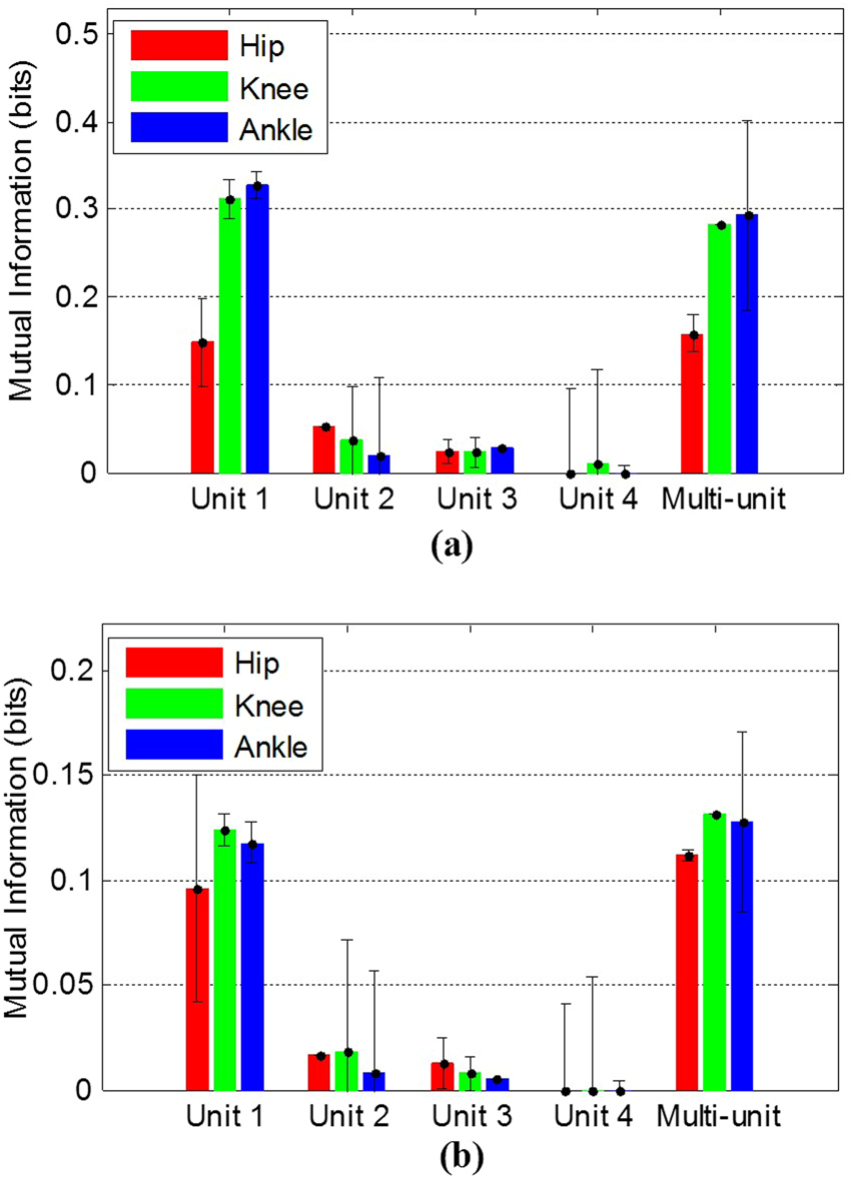
Average (±standard deviation) of mutual information over 31 trials of experiments on five cats: (**a**) Average of MI between the FR of each identified sorted unit activity as well as the FR of multiunit activity and each joint angle; (**b**) Average of MI between the ISI of each identified sorted unit activity as well as the FR of multiunit activity and each joint angle.

The results of MI analysis indicate that single unit activity could provide more information about the limb kinematics than multiunit activity. The average of the MI between FR as well as ISI and joint angles over 31 experimental trials on 5 cats is shown in Fig. 6. The average of the MI between the most informative single-unit FR (ISI) and the hip, knee, and ankle angles are 0.15 ± 0.04, 0.31 ± 0.02, and 0.33 ± 0.02 (0.09 ± 0.05, 0.12 ± 0.01, and 0.12 ± 0.01) respectively, while the average of MI between the multiunit FR (ISI) and the joint angles are 0.16 ± 0.02, 0.28 ± 0.02, and 0.29 ± 0.11 (0.11 ± 0.00, 0.13 ± 0.00, and 0.13 ± 0.05) respectively.

### Decoding Performance

**Figure 7:**
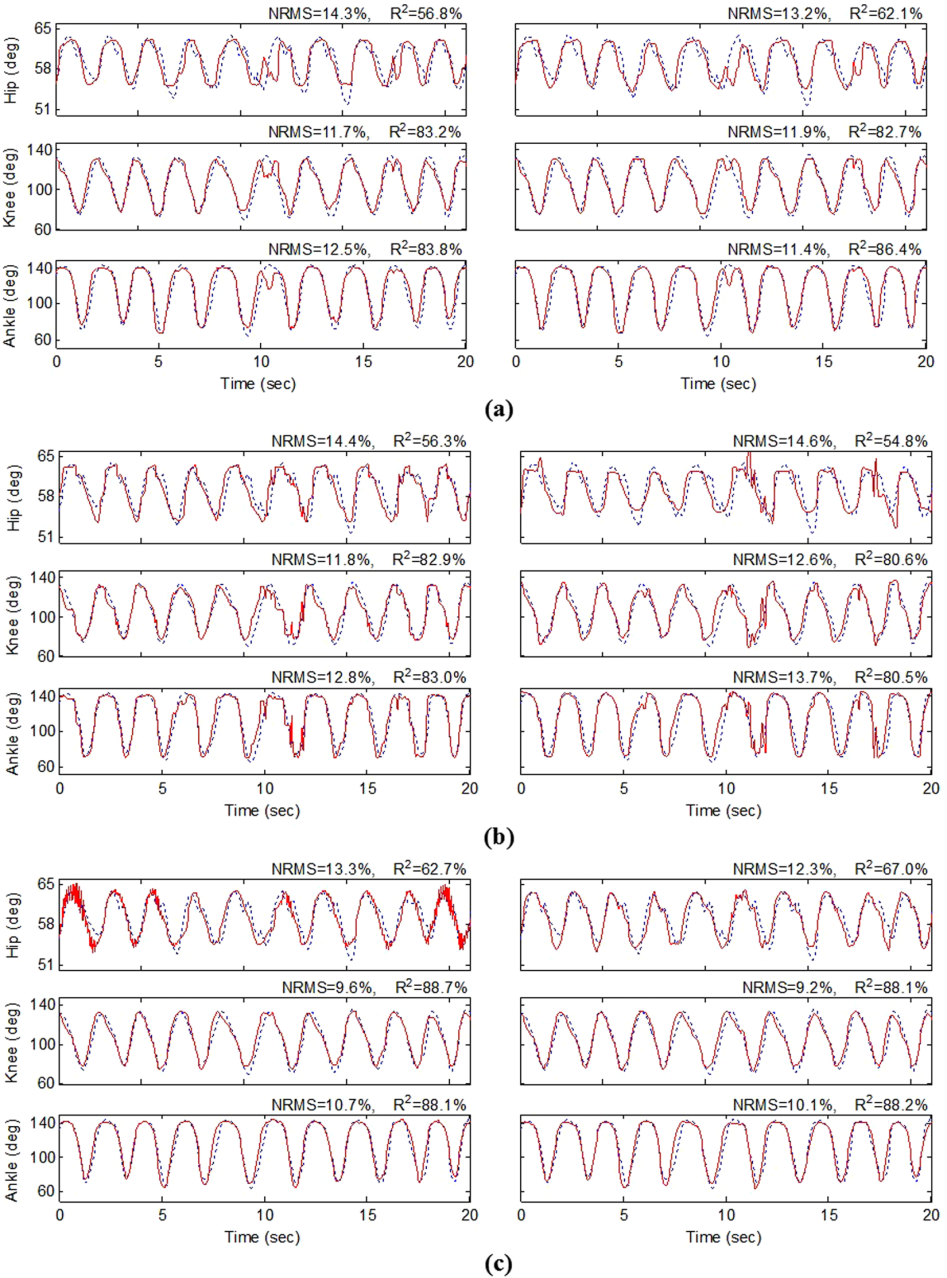
Typical example (trial 5, cat 1) of decoding joint angles: (**a**) using the RNN with the multiunit FR (left) as the input and with the most informative single unit FR as the input (right); (**b**) using the RNN with the multiunit ISI as the input (left) and with the most informative single unit ISI as the input (right); (**c**) using the stacked RNN with the multiunit activity as the input (left) and with the most informative single unit activity (right).

The results of decoding the joint angles show that the decoding performance was significantly improved using the stacked RNN compared to the conventional RNN. However, there is no significant difference between the decoding performance obtained by the single-unit and multiunit activity. Figure 7 illustrates a typical result of decoding the joint angles using different frameworks presented in Methods section. Figure 7(a) shows the results of decoding when the FR of the multiunit activity or the FR of the most informative single unit activity is used as the feature. The average of NRMS decoding error is 12.8% and 12.1% when the FR of the multiunit activity and single unit activity is used as the feature, respectively. For this trial of experiment, the results show that decoding performance is slightly improved (0.7%) when the single unit activity is used for decoding compared to the multiunit activity. Figure 7(b) shows the results of decoding when the ISI of the multiunit activity and of the most informative single unit activity is used as the feature. The average of NRMS decoding error is 13.0% and 13.6% for the multi- and single unit activities, respectively. No improvement in decoding performance is observed for this trial of experiment when the ISI of the single unit activity is used.

Figure 7(c) shows the results when the stacked RNN is used for decoding. The average of NRMS decoding errors are 11.2% and 10.5% for the multi- and single unit activities, respectively. It is observed that the single unit activity provides 0.7% improvement with respect to multiunit activity. The stacked RNN with single unit activity improves the decoding performance by 1.6% compared to when only the FR of the single unit activity is used.

**Figure 8:**
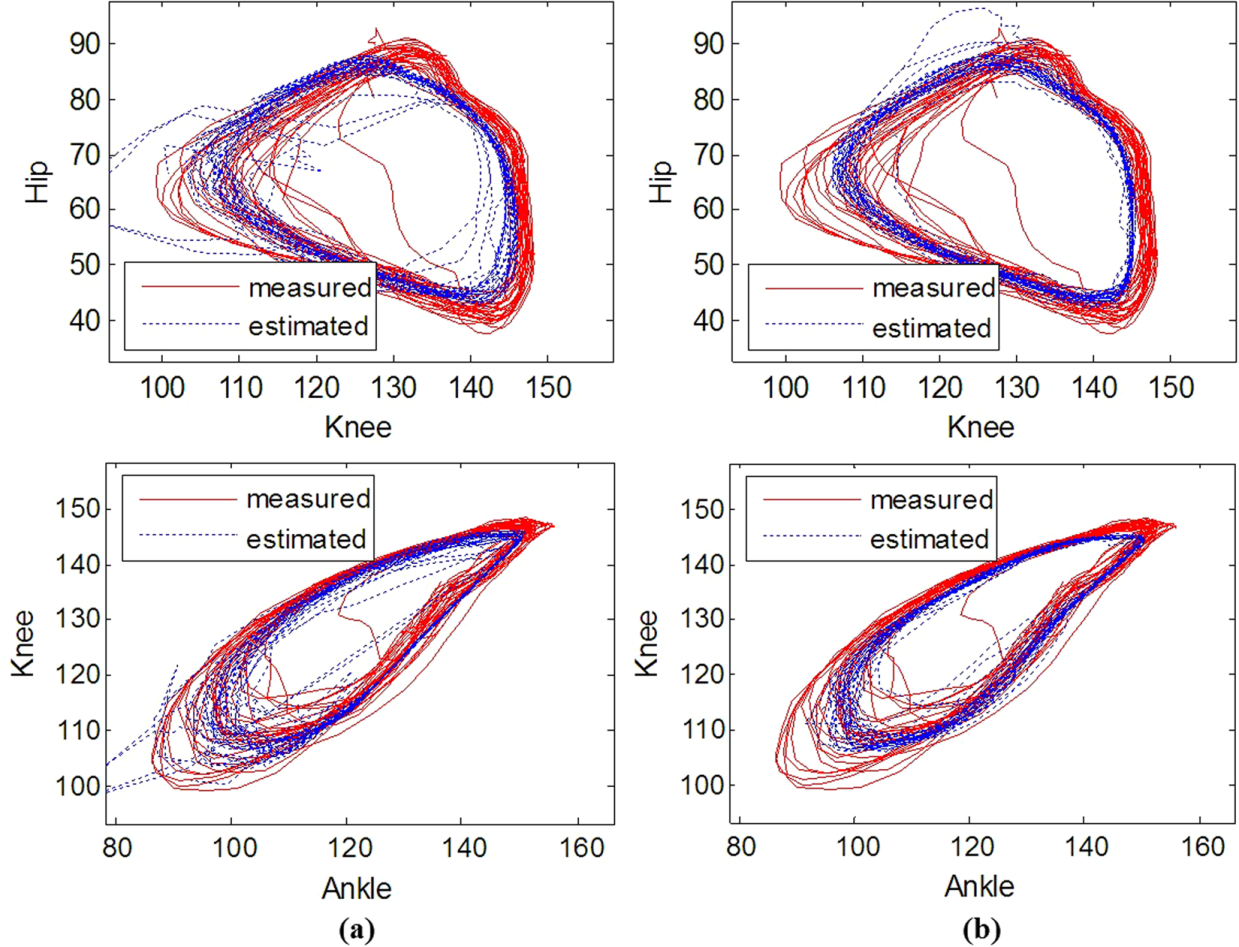
Typical example (cat 1, trial 5) of the angle-angle plot of the estimated and measured joint angle trajectory. The results show for the RNN with the multiunit FR as the input (**a**) and the stacked RNN with the most informative single unit activity (**b**).

Fig. 8 shows the angle-angle plot of the decoding results for the same trial used in Fig. 7. The results indicate that the stacked RNN provides more robust decoding performance compared to the RNN decoder using only FR.

The average of the NRME as well as *R*^2^ over all trials and all animals for each joint angle is summarized in Table 1. The results show that the single unit activity achieves higher decoding performance than the multiunit activity. Also, the stacked RNN based on the FR of the single unit activity achieves higher performance than the RNN. The average of NRMS errors are 11.1%, 11.5%, and 12.9%; and *R*^2^ achieved are 76.8%, 77.8%, and 75.7%, for the hip, knee and ankle joints, respectively, using the stacked RNN with the single unit activities.

**Table 1:** Average of decoding performance over five cats using different methods: RNN with FR as the input, RNN with ISI as the input, and stacked RNN with both FR and ISI as the input.

		Multi-unit		Single unit	
		NRMS%	*R*^2^%	NRMS%	*R*^2^%
FR	hip	11.8 ± 5.1	73.4 ± 24.4	11.2 ± 4.6	76.6 ± 22.7
	knee	12.4 ± 5.0	74.0 ± 23.7	12.5 ± 5.3	72.8 ± 26.1
	ankle	13.1 ± 5.2	75.3 ± 22.2	13.0 ± 5.3	75.2 ± 22.8
	*mean*	12.4 ± 5.1	74.2 ± 22.3	12.3 ± 5.1	74.9 ± 23.6
ISI	hip	17.3 ± 6.7	50.4 ± 33.4	16.3 ± 5.7	54.5 ± 29.5
	knee	16.7 ± 5.2	58.4 ± 26.7	15.9 ± 5.4	59.8 ± 29.5
	ankle	17.2 ± 5.4	59.5 ± 24.2	17.0 ± 5.7	59.9 ± 28.8
	*mean*	17.1 ± 5.8	56.1 ± 28.4	16.4 ± 5.5	58.1 ± 28.8
Stack	hip	11.6 ± 5.3	74.0 ± 27.0	11.1 ± 5.0	76.8 ± 27.2
	knee	12.0 ± 5.3	76.6 ± 25.3	11.5 ± 5.1	77.8 ± 22.5
	ankle	13.1 ± 5.5	75.9 ± 23.8	12.9 ± 5.5	75.7 ± 22.8
	*mean*	12.2 ± 5.3	75.5 ± 25.1	11.8 ± 5.2	76.7 ± 24.0

The results of the two-way analysis of variance (ANOVA) show that there is no significant different in the decoding performance obtained by the multiunit and the single unit activities, but the performance achieved by the stacked RNN is significantly higher than the RNN with FR ($$p=0.093)$$ and higher than the RNN with ISI ($$p=2.760\times {10}^{-15}$$).

### Receptive Fields of Sorted Neurons

**Figure 9:**
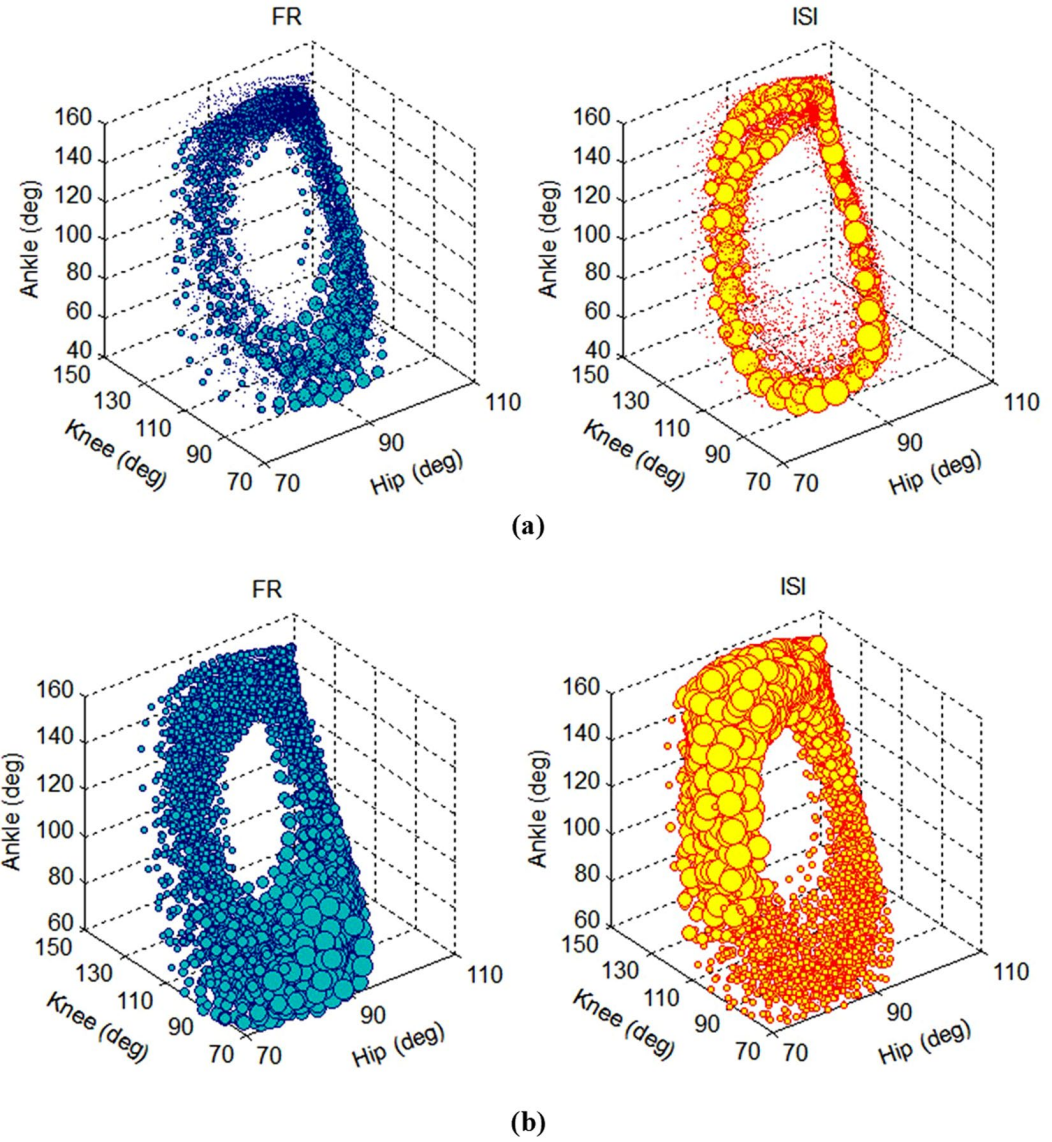
Typical example (cat 1, trial 3) of the receptive fields of the dorsal horn neurons. The response of the least (**a**) and the most informative neurons (**b**) at each position during the passive movement of the limb. The values of FR and ISI at each position have been represented by the spheres which their volumes are proportional to the values of FR or ISI.

To characterize the receptive fields of the dorsal horn neurons, the response of the two sorted neurons; the most and the least informative neurons; is observed at each position of the limb in space. Figure 9 shows the FR and ISI of the two neurons at each position during the passive movement of the limb. The values of FR and ISI at each position have been represented by the spheres which their volumes are proportional to the values of FR or ISI. It can be seen that the least informative neurons (i.e., unit 3) fires spontaneously in the state space (Fig. 9(a)). In contrast, for the most informative unit (i.e., unit 1), the FR increases with increasing the joint angles and decreases with decreasing the joint angles (Fig. 9(b)).

## Discussion

In this work, for the first time, it was demonstrated that hind limb joint angles could be decoded from the dorsal horn recordings using single-electrode recording. It is well known that there are multisegmental connections from one spinal cord level to other levels by the propriospinal fibers. These propriospinal fibers of the cord provide pathways for the multisegmental reflexes that coordinate simultaneous movements in the forelimbs and hind limbs. This fact motivates us to extract whole limb movement (i.e., joint angles) from single-unit recordings. By appropriate signal processing, it should be possible to decompose the information regarding each joint angle from the single-unit recording.

The advantage of dorsal horn over DRG recordings is its convenient accessibility during surgery. Besides, we think that dorsal horn may be a better choice for chronic implantation because it is a compact bulky tissue to place electrodes while DRG is a suspended tissue and previous studies demonstrated that chronic DRG recording did not exceed more than three weeks^2,8^. Moreover, access to the dorsal root ganglia needs more surgical operations and more caution is needed to avoid damaging the tissues during surgery, while the anatomy and structure of dorsal horn makes its accessibility to the surgeon more convenient and less risky.

One concern about dorsal horn recording is the risk of tissue reaction or spinal compression due to the invasive technique applied in this experiment^19^. However, using just one single electrode minimizes this risk. Moreover, the computational cost will be highly reduced in comparison to previous works which one or more microelectrode arrays were used in DRG to record neural signals. To improve the decoding performance using single-electrode recording, RNN with multiple output structure was used. All hind limb joint angles were considered as the outputs of the RNN. To fuse the ISI and FR information for decoding the kinematic information, a stacked generalization approach based on recurrent neural network was proposed. Stacked generalization is a way of constructing ensemble learning combining multiple models to induce a higher-level decoder with improved performance.

Another issue investigated in this study was the role of spike sorting as a preliminary step in extracellular signal processing^25,26^. Based on the statistical test, the results show that there is no significant difference between unsorted spikes and sorted spikes in decoding performance.

The current study just demonstrated the feasibility of the movement kinematic decoding from the neural signal recorded by microelectrode implanted acutely in dorsal horn of anesthetized cats. Estimating the limb position from chronic neural recording in awake animals, investigating the chronic stability and reliability of dorsal horn recording can be considered as a direction for future study.

## References

[1] Ethier C, Miller LE (2015). Brain-controlled muscle stimulation for the restoration of motor function. Neurobiol. Dis..

[2] Weber DJ, Stein RB, Everaert DG, Prochazka A (2007). Limb-state feedback from ensembles of simultaneously recorded dorsal root ganglion neurons. J. Neural Eng..

[3] Stein RB (2004). Coding of position by simultaneously recorded sensory neurones in the cat dorsal root ganglion. J. Physiol..

[4] Wagenaar JB, Ventura V, Weber DJ (2011). State-space decoding of primary afferent neuron firing rates. J. Neural Eng..

[5] Rigosa J, Weber DJ, Prochazka A, Stein RB, Micera S (2011). Neuro-fuzzy decoding of sensory information from ensembles of simultaneously recorded dorsal root ganglion neurons for functional electrical stimulation applications. J. Neural Eng..

[6] Weber DJ, Stein RB, Everaert DG, Prochazka A (2006). Decoding sensory feedback from firing rates of afferent ensembles recorded in cat dorsal root ganglia in normal locomotion. IEEE Trans. Neural Syst. Rehabil. Eng..

[7] Umeda, T. *et al*. Decoding of the spike timing of primary afferents during voluntary arm movements in monkeys. *Front. Neurosci*. **8** (2014). 10.3389/fnins.2014.00097 PMC4023037 24860416

[8] Bruns TM, Wagenaar JB, Bauman MJ, Gaunt RA, Weber DJ (2013). Real-time control of hind limb functional electrical stimulation using feedback from dorsal root ganglia recordings. J. Neural Eng..

[9] Holinski BJ, Everaert DG, Mushahwar VK, Stein RB (2013). Real-time control of walking using recordings from dorsal root ganglia. J. Neural Eng..

[10] Han S, Chu J-U, Kim H, Park JW, Youn I (2017). Multiunit activity-based real-time limb-state estimation from dorsal root ganglion recordings. Sci. Rep..

[11] Han S (2016). An unsorted spike-based pattern recognition method for real-time continuous sensory event detection from dorsal root ganglion recording. IEEE Trans. Biomed. Eng..

[12] Bosco G, Poppele RE (2000). Reference frames for spinal proprioception: kinematics based or kinetics based?. J. Neurophysiol..

[13] Bosco G, Poppele RE (2001). Proprioception from a spinocerebellar perspective. Physiol. Rev..

[14] Bosco G (2003). Modulation of dorsal spinocerebellar responses to limb movement. II. effect of sensory input. J. Neurophysiol..

[15] Bosco G, Eian J, Poppele RE (2006). Phase-specific sensory representations in spinocerebellar activity during stepping: Evidence for a hybrid kinematic/kinetic framework. Exp. Brain Res..

[16] Micera, S. *et al*. Decoding of Grasping Information From Neural Signals Recorded Using Peripheral Intrafascicular Interfaces. *J. Neuroeng. Rehabil*. **8** (2011). 10.1186/1743-0003-8-53 PMC3177892 21892926

[17] Brunton E, Blau CW, Nazarpour K (2017). Separability of neural responses to standardised mechanical stimulation of limbs. Sci. Rep..

[18] Borisoff JF, McPhail LT, Saunders JTW, Birch GE, Ramer MS (2006). Detection and classification of sensory information from acute spinal cord recordings. IEEE Trans. Biomed. Eng..

[19] Im C, Park HY, Koh CS, Ryu SB (2016). Decoding intravesical pressure from local field potentials in rat lumbosacral spinal cord. J. Neural Eng..

[20] Quiroga RQ, Nadasdy Z, Ben-Shaul Y (2004). Unsupervised spike detection and sorting with wavelets and superparamagnetic clustering. Neural Comput..

[21] Tsoi AC, Member S, Back AD (1994). Locally recurrent globally feedforward networks: a critical review of architectures. IEEE Trans. Neural Netw..

[22] Haykin, S. Neural Networks and Learning Machines, Third Editiion. 197–199 (2009).

[23] Zhou, Z.-H. *Ensemble Methods: Foundations And Algorithms*. (CRC press, 2012).

[24] Nicolas-alonso LF, Corralejo R, Gomez-pilar J, Alvarez D, Hornero R (2015). Adaptive stacked generalization for multiclass motor imagery - based brain computer interfaces. IEEE Trans. Neural Syst. Rehabil. Eng..

[25] Gibson S, Judy JW, Markovi D (2012). The first step in decoding the brain. IEEE Signal Process. Mag..

[26] Todorova S, Sadtler P, Batista A, Chase S, Ventura V (2014). To sort or not to sort: the impact of spike-sorting on neural decoding performance. J. Neural Eng..

